# Rapid characterization of CRISPR-Cas9 protospacer adjacent motif sequence elements

**DOI:** 10.1186/s13059-015-0818-7

**Published:** 2015-11-19

**Authors:** Tautvydas Karvelis, Giedrius Gasiunas, Joshua Young, Greta Bigelyte, Arunas Silanskas, Mark Cigan, Virginijus Siksnys

**Affiliations:** Institute of Biotechnology, Vilnius University, Graiciuno 8, LT-02241 Vilnius, Lithuania; DuPont Pioneer, 7300 NW 62nd Avenue, Johnston, IA 50131 USA

**Keywords:** CRISPR, Cas9, Nuclease, PAM determination, Guide RNA determination

## Abstract

To expand the repertoire of Cas9s available for genome targeting, we present a new *in vitro* method for the simultaneous examination of guide RNA and protospacer adjacent motif (PAM) requirements. The method relies on the *in vitro* cleavage of plasmid libraries containing a randomized PAM as a function of Cas9-guide RNA complex concentration. Using this method, we accurately reproduce the canonical PAM preferences for *Streptococcus pyogenes*, *Streptococcus thermophilus* CRISPR3 (Sth3), and CRISPR1 (Sth1). Additionally, PAM and sgRNA solutions for a novel Cas9 protein from *Brevibacillus laterosporus* are provided by the assay and are demonstrated to support functional activity *in vitro* and in plants.

**Electronic supplementary material:**

The online version of this article (doi:10.1186/s13059-015-0818-7) contains supplementary material, which is available to authorized users.

## Background

Cas9 protein of the Type II CRISPR-Cas (Clustered Regularly Interspaced Short Palindromic Repeats-CRISPR associated) [1] bacterial adaptive immune system emerged recently as a promising tool for targeted genome modification in different organisms and cell types [2–5]. Cas9 binds a dual crRNA (CRISPR RNA)-tracrRNA (trans-activating RNA) molecule, or an artificial single-guide RNA (sgRNA) to form a functional complex that acts as an RNA-directed DNA endonuclease capable of generating a DNA double-strand break (DSB) within the target sequence [6, 7]. Cas9 specificity is dictated by the spacer component of the crRNA or sgRNA, which encodes a sequence of approximately 20 nt that hybridizes by direct nucleotide pairings to the complementary strand of the target DNA, the protospacer. Easy programmability of the Cas9 endonuclease using customizable RNAs, should, in theory, enable targeting of any sequence in the genome, however available sequence range is limited by the need of a short nucleotide sequence, termed a protospacer adjacent motif (PAM), that is absolutely required to initiate crRNA-mediated DNA binding [2, 3, 8, 9]. The PAM is usually located in the immediate vicinity of a protospacer sequence complementary to the crRNA and represents a nucleotide signature uniquely associated with each Cas9 protein [6, 7, 10–12].

For genome research and engineering applications where a Cas9-guide RNA system has been successfully reprogrammed to cleave, nick, or bind desired chromosomal DNA targets, typically one system that derived from *Streptococcus pyogenes* (Spy) has been utilized [13–16]. The preferred PAM sequence of Spy Cas9, NGG, constrains targeting to every 8 bp on average in the human genome [6, 17]. If genome specific target sites are desired, the Spy Cas9 PAM requirement additionally limits site selection especially in large complex and partially duplicated plant genomes like maize [18]. Therefore, Cas9 proteins with distinct PAM specificities may help expand the sequence space targeted by Cas9.

In addition to expanding target site density, it is plausible that other Cas9 systems may have unique sequence recognition and enzymatic properties different from those previously described or characterized given the diversity observed between orthologous Cas9 proteins [12, 19]. While cleavage activity and specificity may be enhanced through protein sequence alterations, naturally occurring Cas9s may have different thermodynamic properties which allow conditional regulation. The availability of new Cas9 proteins may also open the way for orthogonal genome engineering allowing different modifications (for example, DNA cleavage and transcriptional activation or silencing) to be performed simultaneously. Moreover, in addition to advancing Cas9 as a new genome research tool, the characterization of new Cas9 proteins and establishment of their associated biochemical properties should contribute to our understanding of structure-function relationships in bacterial adaptive immunity cascade.

With >1000 Cas9 sequences available in sequence databases and the continued sequencing of microbial genomes becoming routine [2, 19], Cas9 orthologues are abundant. However, methods to ascertain the PAM sequence requirement for new Cas9 proteins are limited. Typically, PAM sequences of new Cas9 proteins are identified by bioinformatic analysis of sequences immediately flanking putative protospacers in bacteriophage genomes [20]. With most of the spacers in available Type II CRISPR arrays exhibiting only a few if any matches to available phage sequences, this approach constrains the exploration of Cas9 protein diversity for genomic applications.

To tap into this unexplored diversity and expand the repertoire of Cas9s available for genome targeting applications, the development of a method that allows the direct read-out of Cas9 endonuclease PAM specificity as a function of Cas9-guide RNA complex concentration *in vitro* is reported. Briefly, plasmid DNA vectors containing a unique spacer sequence juxtaposed to random PAM libraries are subjected to digestion *in vitro* using purified Cas9 protein and guide RNA complexes. The digested products are captured by linker addition, and subjected to PCR amplification and sequencing to identify PAMs recognized by Cas9. The canonical PAM sequences for *Streptococcus pyogenes* (Spy), *Streptococcus thermophilus* CRISPR1 (Sth1), and *Streptococcus thermophilus* CRISPR3 (Sth3) are confirmed and the PAM sequence and guide RNA for an uncharacterized Cas9 from *Brevibacillus laterosporus* SSP360D4 (Blat) are identified. Using the novel PAM and guide RNA solutions from the described assays, experimental evidence is also provided for Blat Cas9 functional activity both *in vitro* and in plants. The methods described here pave the way for the characterization of novel Cas9 proteins opening the door to a new era of genome modification with orthologous Cas9-guide RNA systems.

## Results

### Design and construction of randomized libraries for assaying Cas9 PAM preferences

**Fig. 1:**
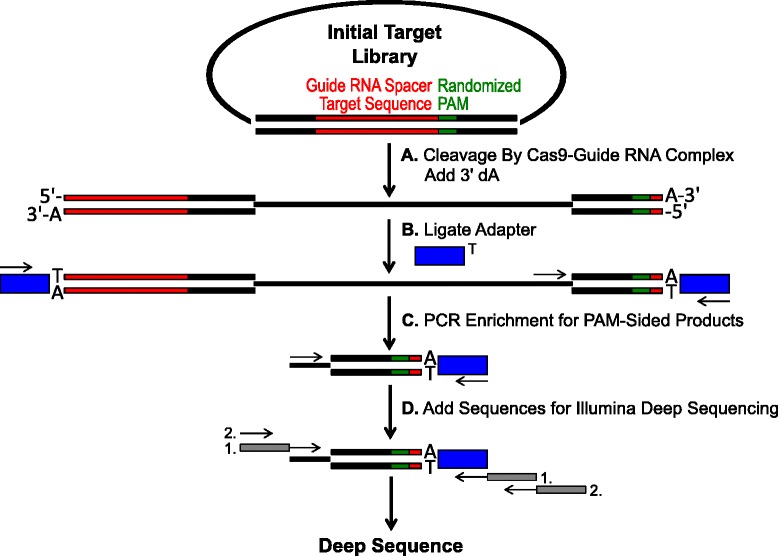
Schematic for identification of PAM preferences by Cas9 cleavage *in vitro*. **a** Initial plasmid library with randomized PAM (green box) is cleaved with Cas9 complex and 3′ dA overhangs are added. **b** Adapters with 3′ dT overhang (blue box) are ligated to both ends of the cleavage product. **c** Primers are utilized to enrich for PAM-sided cleaved products by PCR. **d** After PCR enrichment, DNA fragments are purified and Illumina compatible anchors and barcodes are ‘tailed-on’ through two rounds of PCR (gray boxes) and Illumina deep sequenced

PAM libraries containing randomized DNA sequences immediately downstream of a DNA sequence complementary to the spacer of a guide RNA were generated and used to empirically determine the PAM recognition of Type II Cas9 endonucleases (Fig. 1). With the guide RNA spacer target sequence being fixed, the randomized bases serve as a substrate for the direct read-out of Cas9 endonuclease PAM specificity. Randomized sequences were introduced into a plasmid DNA vector in the PAM region of a protospacer target sequence demonstrating perfect homology to the guide RNA spacer T1 (CGCUAAAGAGGAAGAGGACA). Two libraries increasing in size and complexity from five randomized base pairs (1,024 potential PAM combinations) to seven randomized base pairs (16,384 potential PAM combinations) were generated. Randomization of the 5 bp library was introduced through the synthesis of a single oligonucleotide containing five random residues. The single-stranded oligonucleotide was converted into a double-stranded template by PCR (Additional file 1: Figure S1A), cloned into the plasmid vector (Additional file 1: Figure S1B) and transformed into *E. coli* as described in the Methods section. To ensure optimal randomness in the 7 bp PAM library, the size and complexity of the library was reduced by synthesizing four oligonucleotides each containing six random residues plus a seventh fixed residue comprising G, C, A, or T, respectively. Each of the four oligonucleotides were separately converted into double-stranded DNA, cloned into vector pTZ57R/T as described in the Methods section and transformed into *E. coli* as described for the 5 bp library. After transformation, plasmid DNA was recovered and combined from each of the four 6 bp PAM libraries to generate a randomized 7 bp PAM library comprising 16,384 possible PAM combinations. For both libraries, incorporation of randomness was validated by deep sequencing; examining the nucleotide composition at each position of the PAM region using a position frequency matrix (PFM) (Methods section and [21]) (Additional file 1: Figure S2A and B). The distribution and frequency of each PAM sequence in the 5 bp and 7 bp randomized PAM library are shown in Additional file 1: Figures S3 and S4, respectively.

### Assaying Cas9 PAM preferences

The randomized PAM libraries described in the previous section were subjected to *in vitro* digestion with different concentrations of recombinant Cas9 protein preloaded with guide RNA in order to assay Cas9 endonuclease PAM preferences in a dose-dependent manner. After digestion with Cas9-guide RNA ribonucleoprotein (RNP) complexes, PAM sequence combinations from the randomized PAM library that supported cleavage were captured by ligating adapters to the free-ends of the plasmid DNA molecules cleaved by the Cas9-guide RNA complex (Fig. 1a and b). To promote efficient ligation and capture of the cleaved ends, the blunt-ended double-stranded DNA cut generated by Cas9 endonucleases [6, 7, 22] was modified to contain a 3′ dA overhang and adapters were modified to contain a complementary 3′ dT overhang. To generate sufficient quantities of DNA for sequencing, DNA fragments harboring the PAM sequence supporting cleavage were PCR amplified using a primer in the adapter and another directly adjacent to the PAM region (Fig. 1c). The resulting PCR amplified Cas9 PAM libraries were converted into ampli-seq templates (Fig. 1d) and single-read deep sequenced from the adapter-side of the amplicon. To ensure adequate coverage, the Cas9 PAM libraries were sequenced to a depth at least five times greater than the diversity in the initial randomized PAM library (5,120 and 81,920 reads for the 5 and 7 bp PAM randomized libraries, respectively). PAM sequences were identified from the resulting sequence data by only selecting those reads containing a perfect 12 nt sequence match flanking either side of the 5 or 7 nt PAM sequence (depending on the randomized PAM library used); capturing only those PAM sequences resulting from perfect Cas9-guide RNA target site recognition and cleavage. To compensate for inherent bias in the initial randomized PAM libraries, the frequency of each PAM sequence was normalized to its frequency in the starting library. Since the assay described here directly captures Cas9 cleavable PAM sequences, probabilistic modeling was used to calculate the PAM consensus for each Cas9 protein. This was accomplished by evaluating the probability of finding each nucleotide (G, C, A, or T) at each position of the PAM sequence independently using a position frequency matrix (PFM) (Methods section and [21]). The resulting probabilities were then visualized as a WebLogo [23].

To examine the propensity for false positives in the assay, the addition of Cas9 RNP complexes in the digestion step was omitted (Fig. 1a) and the assay was performed through the PCR enrichment step (Fig. 1c). As shown in Additional file 1: Figure S5A, no amplification products were detected in the absense of Cas9-guide RNA complexes. Thus, indicating that the incidence of false positives is low and does not significantly contribute to results of the assay.

### PAM preferences of *Streptococcus pyogenes* and S*treptococcus thermophilus* (CRISPR3 and CRISPR1 systems) Cas9 proteins

**Fig. 2:**
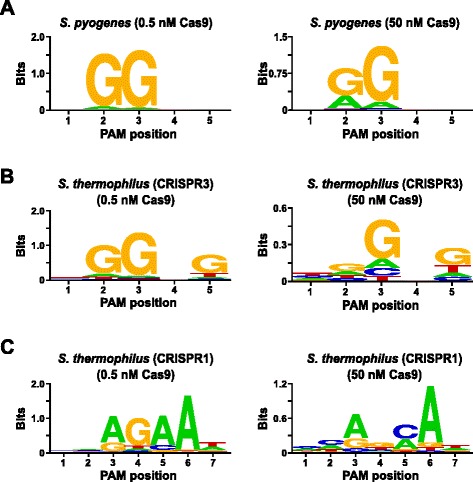
PAM preferences for *S. pyogenes* (**a**), *S. thermophilus* CRISPR3 (**b**), and *S. thermophilus* CRISPR1 (**c**) Cas9 proteins. Frequency of nucleotides at each PAM position was independently calculated using a position frequency matrix (PFM) [21] and plotted as a WebLogo [23]

In order to validate the assay, the PAM preferences of *Streptococcus pyogenes* (Spy) and *Streptococcus thermophilus* CRISPR3 (Sth3) Cas9 proteins, whose PAM sequence requirement have been previously reported [6, 7, 10, 24], were examined. *In vitro* digests were carried out with 1 μg (5.6 nM) of the 5 bp randomized PAM library at two concentrations, 0.5 and 50 nM, of pre-assembled Spy or Sth3 Cas9 protein, crRNA, and tracrRNA RNP complexes [6, 7, 25] for 1 h in a 100 μL reaction volume. Based on their frequency in the 5 bp randomized PAM library, Spy and Sth3 Cas9 PAM sequences (NGG and NGGNG, respectively) were at final concentrations of 0.40 nM and 0.11 nM in the digestion, respectively. Members of the randomized PAM library that contained PAM sequences which supported cleavage were captured and identified as described in the previous section. As a negative control, the starting uncleaved randomized PAM library was subject to sequencing and PFM analysis alongside those libraries exposed to Cas9 RNP complexes. As shown in Additional file 1: Figure S5B and C, no sequence preferences exist in the absence of Cas9 RNP complex digestion as evident by a near perfect distribution of each nucleotide at each position of the PAM in the PFM table and the lack of informative content in the WebLogo for the control. This is in stark constrast with Fig. 2a and b that illustrates the composition of the sequences derived from libraries digested with Spy and Sth3 Cas9 RNP complexes. Examination of the PFM derived WebLogos (Fig. 2a and b) also reveal the presence of the canonical PAM preferences for the Spy and Sth3 Cas9 proteins, NGG [6] and NGGNG [7, 10, 24], respectively. Although the PAM preferences reported for Spy and Sth3 Cas9 proteins are observed in both the 0.5 nM and 50 nM digests, there is a general broadening in specificity under the 50 nM digest conditions. This is most evident at position 2 for the Spy Cas9 protein where the frequency of a non-canonical A residue increases dramatically (Fig. 2a). For Sth3, all PAM positions exhibit a marked decrease in specificity as a result of increasing the RNP complex concentration (Fig. 2b).

Further validation of the assay was conducted by examining the PAM preferences for the *Streptococcus thermophilus* CRISPR1 (Sth1) Cas9 protein whose PAM specificity has been reported to extend out to 7 bp [10, 12]. Using 1 μg (5.6 nM) of the 7 bp randomized PAM library as template, Sth1 Cas9-guide RNA digestions were carried out at two concentrations, 0.5 nM and 50 nM, of RNP complex as described above. As controls, Spy and Sth3 Cas9 RNP complexes were also used to digest the 7 bp randomized PAM library but only at single, 0.5 nM, RNP complex concentration. Based on the frequency in the 7 bp randomized PAM library, the PAM sequences previously reported for Sth1 (NNAGAAW), Spy (NGG), and Sth3 (NGGNG) were at final concentrations of 0.01 nM, 0.22 nM, and 0.05 nM, respectively. As shown in Additional file 1: Figure S6A and B, the PAM preferences for Spy and Sth3 Cas9 proteins generated using the 7 bp library were nearly identical to those produced with the 5 bp library providing strong evidence for the reproducibility of the assay. The PAM preferences for the Sth1 Cas9 protein also closely matched that previously reported, NNAGAAW [10], at the 0.5 nM Cas9-guide RNA complex concentration (Fig. 2c). Similar to Spy and Sth3 Cas9 proteins, Sth1 Cas9 was capable of cleaving a more diverse set of PAM sequences in the reactions containing a higher concentration of Cas9-guide RNA complex (50 nM), the most striking was the marked loss of the G residue requirement at position 4 and the near equal preference for a C and A bp at position 5 (Fig. 2c). This resulted in a different PAM consensus than that obtained at lower concentrations.

To examine whether PAM specificity is independent of the type of guide RNA, duplexed crRNA:tracrRNA or sgRNA [6, 7], Spy, Sth3, and Sth1 Cas9 PAM preferences were also examined using a binary Cas9 and sgRNA RNP complex. Digestion was carried out at a single RNP complex concentration of 0.5 nM and PAM preference analysis was performed as described above. As shown in Additional file 1: Figure S7A, B, and C, PAM preferences were nearly identical regardless of the type of guide RNA used; either a crRNA:tracrRNA duplex or sgRNA. In addition, to confirm that PAM specificity is not greatly influenced by the composition of the target DNA or spacer sequence, the sequence on the opposite side of the 5 or 7 bp randomized library was targeted for cleavage with a different spacer; T2-5 (UCUAGAUAGAUUACGAAUUC) for the 5 bp library or T2-7 (CCGGCGACGUUGGGUCAACU) for the 7 bp library. Spy and Sth3 Cas9 proteins preloaded with sgRNAs targeting the T2 sequence were used to interrogate the 5 bp randomized PAM library while the Sth1 Cas9-T2 sgRNA complexes were used to digest the 7 bp randomized PAM library. PAM preferences were assayed as described above. The PAM preferences for all 3 Cas9 proteins were nearly identical regardless of spacer and target DNA sequence (Additional file 1: Figure S8A, B, and C).

### Identification of sgRNA and PAM preferences for the *Brevibacillus laterosporus* Cas9 protein

**Fig. 3:**
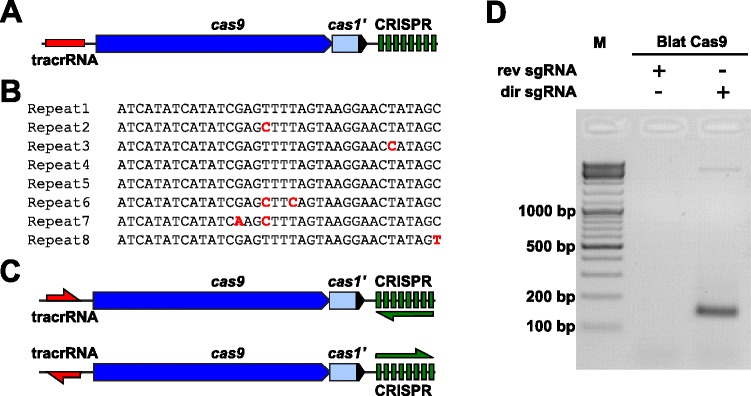
Identification of Type II CRISPR-Cas elements in *Brevibacillus laterosporus* SSP360D4 CRISPR-Cas system. **a** An illustration of the genomic DNA region from the Type II CRISPR-Cas system from *Brevibacillus laterosporus* SSP360D4. **b** Comparison of Type II CRISPR array repeat sequences identified in *Brevibacillus laterosporus* SSP360D4*.* **c** The ‘direct’ and ‘reverse’ tracrRNA and CRISPR array transcriptional scenarios for the Type II CRISPR-Cas system from *Brevibacillus laterosporus* SSP360D4. **d** An agarose gel with reaction products, indicating that only the ‘direct’ sgRNA (dir sgRNA), but not the ‘reverse’ sgRNA (rev sgRNA) support plasmid library cleavage in combination with the Cas9 endonuclease originating from *Brevibacillus laterosporus* SSP360D4

To empirically examine the PAM preferences for a Cas9 protein whose PAM was undefined, an uncharacterized Type II-C CRISPR-Cas locus from *Brevibacillus laterosporus* strain SSP360D4 (Blat) was identified by searching internal DuPont Pioneer databases for Cas9 orthologues. The locus (approximately 4.5 kb) contained a *cas9* gene capable of encoding a 1,092 polypeptide, a CRISPR array comprising seven repeat-spacer units just downstream of the *cas9* gene and a tracrRNA encoding region located upstream of the *cas9* gene with partial homology to the CRISPR array repeats (Fig. 3a). The repeat and spacer length (36 and 30 bp, accordingly) is similar to other Type II CRISPR-Cas systems with five of the eight repeats containing 1 or 2 bp mutations (Fig. 3b and Additional file 1: Figure S9). Other genes typically found in a Type II CRISPR-Cas locus were either truncated (*cas1*) or missing (Fig. 3a).

The guide RNA requirement for the Blat Cas9 protein was determined by generating two sgRNA variants. These variants were generated to account for both possible sense or anti-sense expression scenarios of the tracrRNA and CRISPR array (Fig. 3c) and used to probe which expression scenario supported cleavage activity of Blat Cas9 in the randomized PAM library. Single guide RNAs were designed by first identifying the boundaries of the putative tracrRNA molecules by analyzing regions which were partially complementary to the 22 nt 5′ terminus of the repeat (anti-repeat). Next, to determine the 3′ end of the tracrRNA, possible secondary structures and terminators were used to predict the region of termination in the downstream fragment. This was accomplished by screening for the presence of Rho independent-like termination sequences in the DNA surrounding the anti-repeat similar to that described in Karvelis et al. [25], converting the surrounding DNA into RNA sequence and examining the resulting structures using UNAfold [26]. The resultant sgRNAs were designed to contain a T7 polymerase transcription initiation recognition signal at the 5′ end followed by a 20 nt target recognition sequence, 16 nt of crRNA repeat, 4 nt self-folding hairpin loop, and anti-repeat sequence complementary to the repeat region of the crRNA followed by the remaining 3′ part of the putative tracrRNA. The sgRNA variant which contains a putative tracrRNA transcribed in the same direction as the *cas9* gene (Fig. 3c) is termed ‘direct’ sgRNA, while the sgRNA containing the tracrRNA transcribed in the opposite direction a ‘reverse’ sgRNA. Fifty nM of Blat Cas9 sgRNA RNP complex, pre-loaded with either the ‘direct’ or ‘reverse’ sgRNAs, respectively, were incubated with 1 μg (5.6 nM) of the 7 bp randomized PAM library. After library digestion and addition of 3′ dA overhangs, adapters were ligated and cleavage products were PCR amplified (Fig. 1). Analysis of reaction products by agarose gel electrophoresis revealed that the ‘direct’ sgRNA, but not the ‘reverse’ sgRNA supported plasmid library cleavage (Fig. 3d). The sequence and predicted secondary structure of the ‘direct’ sgRNA are shown in Additional file 1: Figure S10.

**Fig. 4:**
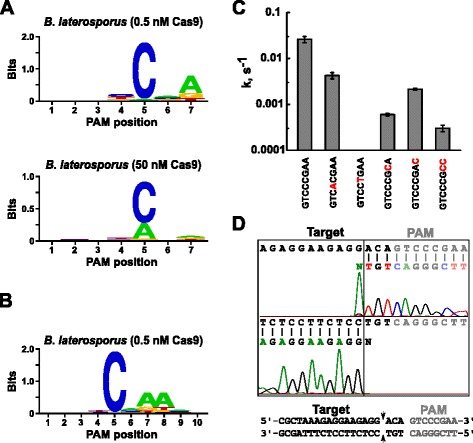
PAM preferences and cleavage positions of *Brevibacillus laterosporus* SSP360D4 (Blat) Cas9 enzyme. Blat Cas9 PAM preferences when 1 μg of library DNA was cleaved with 0.5 nM or 50 nM Cas9-sgRNA complex (**a**), extended out to position 10 by shifting the protospacer target by 3 bp (**b**). Frequency of nucleotides at each PAM position was independently calculated using a position frequency matrix (PFM) [21] and plotted as a WebLogo [23]. **c** Cleavage rates of supercoiled plasmid DNA substrates containing mutations (shown in red) in GTCCCGAA PAM sequence. All data points are mean values from ≥3 independent experiments. Error bars are given as S.D. **d** Run-off sequencing from both sense and anti-sense directions of plasmid DNA cleaved with Blat Cas9

After determining the appropriate guide RNA for Blat Cas9, PAM identification was performed similarly to that described above for the Spy, Sth3, and Sth1 Cas9 proteins against the 7 bp randomized PAM library with two concentrations, 0.5 and 50 nM, of pre-assembled Blat Cas9 ‘direct’ sgRNA RNP complex. As shown in Fig. 4a, the PFM WebLogo PAM consensus for the Blat Cas9 protein under the 0.5 nM digest conditions was NNNNCND (N = G, C, A, or T; D = A, G, or T) with a strong preference for a C at position 5 of the PAM sequence. A moderate preference for an A was observed at position 7 and slight preferences for a C or T at position 4 and G, C, or A over T at position 6 were also noted when closely examining the PFM table (Additional file 1: Figure S11). Similarly to Spy, Sth3, and Sth1 Cas9 proteins, the PAM specificity broadens as the Cas9-sgRNA complex concentration increases. This is most evident at position 5 where a larger proportion of PAM sequences containing an A residue support cleavage at 50 nM compared with the 0.5 nM digest conditions.

Since Blat Cas9 may accept any base in the first three positions of its PAM sequence (Fig. 4a), the spacer T1 was shifted by three nucleotides in the 5′ direction to allow PAM identification to be extended from 7 to 10 bp. The shifted T1 spacer, T1-3 (AAACGCUAAAGAGGAAGAGG), was incorporated into the Blat ‘direct’ sgRNA and PAM identification was performed as described previously for Spy, Sth3, Sth1, and Blat Cas9 proteins. PAM preference analysis revealed the PAM specificity for Blat Cas9 may be extended out to position 8 where there is a moderate preference for an additional A (Fig. 4b).

PAM specificity for Blat Cas9 was confirmed by generating plasmids to contain mutations in the most conserved residues of the PAM (Fig. 4c). Replacement of the C nucleotide at position 5 abolished plasmid DNA cleavage confirming its key role in Blat Cas9 PAM recognition. Replacement of A nucleotides at positions 7 and 8 significantly reduced (43× and 12×, respectively) the cleavage rate of supercoiled plasmid also indicating the importance of these nucleotides in Blat Cas9 PAM recognition.

To identify the DNA target cleavage positions for the Blat Cas9 protein, a plasmid containing a 20 bp region matching the spacer T1 followed by a PAM sequence, GTCCCGAA, falling within the PAM consensus for Blat Cas9, NNNNCNDD, was generated and digested with Blat Cas9-guide RNA ribonucleoprotein complex. Direct DNA sequencing was used to determine the ends of the linear DNA molecule generated by the Blat Cas9 RNP complex. The sequence results confirmed that plasmid DNA cleavage occurred in the protospacer 3 nt 5′ of the PAM sequence (Fig. 4d) similar to that observed for Spy, Sth3, and Sth1 Cas9 proteins [6, 7, 22].

### *In planta* genome editing using Blat Cas9 and sgRNA

Following elucidation of the sgRNA and PAM preferences for Blat Cas9, maize optimized Cas9 and sgRNA expression cassettes were generated for *in planta* testing as previously described for the *S. pyogenes cas9* gene and sgRNA [27]. Briefly, the Blat *cas9* gene was maize codon optimized and intron 2 of the potato *ST-LSI* gene was inserted to disrupt expression in *E. coli* and facilitate optimal splicing *in planta* [28] (Additional file 1: Figure S12). Nuclear localization of the Blat Cas9 protein in maize cells was facilitated by the addition of both amino and carboxyl-terminal nuclear locations signals, SV40 (MAPKKKRKV) and *Agrobacterium tumefaciens* VirD2 (KRPRDRHDGELGGRKRAR), respectively (Additional file 1: Figure S12). The Blat *cas9* gene was constitutively expressed in plant cells by linking the optimized *cas9* to a maize Ubiquitin promoter [29] and pinII terminator [30] in a plasmid DNA vector. To confer efficient sgRNA expression in maize cells, a maize U6 polymerase III promoter and terminator (TTTTTTTT) were isolated and fused to the 5′ and 3′ ends of a modified Blat sgRNA encoding DNA sequence, respectively (Additional file 1: Figure S13). The modified Blat sgRNA contained two modifications from that used in the *in vitro* studies; a T to G alteration at position 99 and a T to C modification at position 157 of the sgRNA (Additional file 1: Figure S13). The changes were introduced to remove potential premature U6 polymerase III termination signals in the Blat sgRNA. Alterations where introduced to have minimal impact on the secondary structure of the sgRNA compared to the version used in the *in vitro* studies (data not shown).

To accurately compare the mutational efficiency resulting from the imperfect non-homologous end-joining (NHEJ) repair of DNA double-strand breaks (DSBs) resulting from Spy and Blat Cas9 cleavage, protospacer identical genomic target sites were selected by identifying targets with Spy and Blat Cas9 compatible PAMs, NGGNCNDD. Identical spacer sequences were selected for Blat and Spy Cas9 by capturing the 18 to 21 nt sequence immediately upstream of the PAM. To ensure optimal U6 polymerase III expression and not introduce a mismatch within the sgRNA spacer, all target sequences were selected to naturally terminate in a G at their 5′ end. Targets were identified and selected in exon 1 and 4 of the maize fertility gene *Ms45* and in a region upstream of the maize *liguleless-1* gene.

**Fig. 5:**
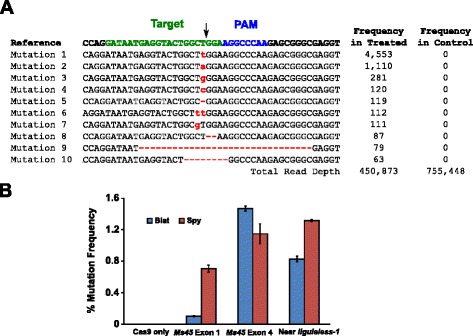
*Brevibacillus laterosporus* Cas9 promotes NHEJ mutations in maize. **a** Top 10 most prevalent types of NHEJ mutations detected with Blat Cas9 in exon 4 of the *Ms45* gene. A black arrow indicates the expected site of cleavage; mutations are highlighted in red; lower case font indicates an insertion; ‘-’ indicates a deletion. **b** Comparison of Spy and Blat Cas9 NHEJ mutation frequencies at three protospacer identical target sites in maize. NHEJ mutations were detected by deep sequencing 3 days after transformation. Error bars represent SEM, n = 3 particle gun transformations. Cas9 only is the negative control and represents the average (across all three target sites) background frequency of mutations resulting from PCR amplification and sequencing

The mutational activity of Blat Cas9 in maize was examined by biolistically transforming 10-day-old immature maize embryos (IMEs) with DNA vectors containing *cas9* and sgRNA genes. Blat and the equivalent Spy Cas9 and sgRNA expression vectors were independently introduced into maize Hi-Type II [31] IMEs by particle gun transformation similar to that described in [27, 32]. Since particle gun transformation can be highly variable, a visual marker DNA expression cassette, Ds-Red, was also co-delivered with the Cas9 and sgRNA expression vectors to aid in the selection of evenly transformed IMEs. In total, three transformation replicates were performed on 60–90 IMEs and 20–30 of the most evenly transformed IMEs from each replicate were harvested 3 days after transformation. Total genomic DNA was extracted and the region surrounding the target site was amplified by PCR and amplicons sequenced to a read depth in excess of 300,000. The resulting reads were examined for the presence of mutations at the expected site of cleavage by comparison to control experiments where the sgRNA DNA expression cassette was omitted from the transformation. As shown in Fig. 5a, mutations were observed at the expected site of cleavage for Blat Cas9 with the most prevalent types of mutations being single base pair insertions or deletions. Similar repair patterns were also observed for the Spy Cas9 protein (Additional file 1: Figure S14 and [27]). The mutational activity for Blat Cas9 was robust at two of the three sites tested and exceeded that of the Spy Cas9 at the *Ms45* exon 4 target site by approximately 30 % (Fig. 5b).

## Discussion

The DNA target site for Cas9 is composite and consists of a protospacer sequence and a short PAM sequence adjacent to the protospacer. Target recognition is achieved through a complex mechanism involving Cas9-mediated interaction with the PAM and crRNA-guided interactions with the complementary DNA of the protospacer [8, 9]. The process initiates with PAM recognition by Cas9 and then proceeds through crRNA-guided sequence-specific hybridization with a protospacer [33]. In this respect, the PAM sequence plays a key role in target recognition by licensing crRNA-guided base pairing to the protospacer sequence [8, 9]. A strict PAM requirement constrains DNA target selection and poses a limit to Cas9 genome editing applications. Target site selection may be further confined if unique genomic sites are required especially in large complex plant genomes like maize [18]. These constraints imposed by the PAM and the specificity of the Spy Cas9 could be overcome either by systematically redesigning the PAM specificity of a single Cas9 protein [34], by simply exploring the natural diversity of Cas9 proteins or by combining the two approaches.

In addition to expanding the sequence space targeted by Cas9, orthologous Cas9 proteins with different biochemical activities may enhance genomic manipulation efforts. Cas9 systems with enhanced specificity or tunable activity may help mitigate off-target concerns while systems with incompatible guide RNAs or PAM sequences could be used to concertedly edit, activate, or repress different targets. Thus, by combining these features unique control over genome expression and content may be afforded.

To facilitate the rapid expansion of the RNA-guided Cas9 toolkit, a method was developed to empirically examine the PAM sequence requirements for any Cas9 protein. The method is based on the analysis of the *in vitro* cleavage products of a plasmid DNA library which contains a fixed protospacer target sequence and a stretch of five or seven randomized base pairs in the putative PAM region. Using this method, the canonical PAM preferences for Cas9 proteins of *S. pyogenes* and *S. thermophilus* CRISPR1 and CRISPR3 systems were confirmed. Next, the method was applied to an uncharacterized Cas9 protein from the Type II CRISPR-Cas system of *B. laterosporus* SSP360D4*.* In the Type II system of *B. laterosporus*, the transcriptional direction of the tracrRNA and CRISPR region could not be reliably predicted by computational approaches. Therefore, two single guide RNA (sgRNA) variants for both possible sense and anti-sense expression scenarios of the tracrRNA and CRISPR array (Fig. 3c) were synthesized. The randomized PAM library was then used to test which expression scenario (sgRNA) supported cleavage. With a functional sgRNA identified, analysis of the cleavage products from the 7 bp randomized PAM library revealed a novel PAM requirement for the *B. laterosporus* Cas9. One that requires a strong preference for a C residue at position 5 of the PAM sequence followed by moderate preferences for A residues at positions 7 and 8 with an overall PAM consensus of NNNNCNDD (N = G, C, A, or T; D = A, G, or T). With a strong preference for just a single nucleotide, *B. laterosporus* Cas9 provides a useful addition to the Cas9 toolbox.

To examine the robustness of the assays established here, the *B. laterosporus* SSP360D4 Cas9 and sgRNA were tested in maize. As a result of cleavage, imperfect DNA repair resulted in INDEL mutations at all three chromosomal sites tested with robust INDEL frequencies observed at two of the three sites. Interestingly, at one of the sites, an enhancement in the recovery of INDEL mutations of approximately 30 % was observed for the *B. laterosporus* Cas9 over the *S. pyogenes* Cas9.

Importantly, this *in vitro* assay also revealed that cleavage of permissive PAMs is dependent on Cas9 concentration. For all Cas9 proteins analyzed, PAM sequences licensing plasmid DNA cleavage at higher (50 nM) Cas9-guide RNA complex concentrations are more relaxed than PAM sequences identified at low (0.5 nM) Cas9-guide RNA complex concentrations. This finding corroborates previous studies which demonstrated that lowering Cas9 concentration and shortening cleavage time prevents off-target cleavage by *S. pyogenes* Cas9 in vivo [35, 36]. Additionally, most other PAM determination methods have been performed in cells or cell extracts by expressing Cas9 at undefined concentrations [34, 37–39]. Given this, the Cas9 PAM recognition results from these studies may be difficult to accurately interpret. A case in point is reflected in the inability of previous attempts [12, 37, 39] to precisely reproduce the PAM recognition of *S. thermophilus* CRISPR1 (Sth1) Cas9 protein originally reported by [10] while the methods described here accurately recapitulate the PAM recognition of Sth1 Cas9 albeit at lower Cas9-guide RNA ribonucleoprotein complex concentrations. Taken together, the methods established here further refine PAM specificity assessments by the dose-dependent control of recombinant Cas9 protein *in vitro* enabling an accurate detailed examination of Cas9 PAM recognition as a function of Cas9 and guide RNA complex concentration.

## Conclusions

The assays described here further refine Cas9 PAM discovery efforts by the use of recombinant Cas9 protein and reframe PAM specificity as being non-static and dependent on Cas9-guide RNA complex concentration. Proof of concept for the described methods is provided by identifying the PAM preferences of a novel Cas9 protein from *B. laterosporus* SSP360D4 and by demonstrating its functional activity in maize. These methods pave the way for the development of novel Cas9-based tools for next generation of genome editing applications.

## Methods

### Oligonucleotides

Sequences of all oligonucleotides and primers used in this study are listed in Additional file 1: Table S1.

### Cloning of *cas9* genes

The *cas9* genes of the CRISPR3-Cas system of *Streptococcus thermophilus* (Sth3), the CRISPR1-Cas system of *Streptococcus thermophilus* (Sth1), and *Brevibacillus laterosporus* (Blat) were amplified directly from a genomic DNA samples, while the *cas9* gene of *Streptococcus pyogenes* (Spy) from the plasmid, pMJ806 (a gift from Jennifer Doudna (Addgene plasmid # 39312)) using Sth3-dir/Sth3-rev, Sth1-dir/Sth1-rev, Blat-dir/Blat-rev and Spy-dir/Spy-rev primers pairs accordingly, and ligated into a pBAD24-CHis expression vector [40] digested over NcoI and XhoI sites.

### Expression and purification of Cas9 proteins

Sth1, Sth3 and Spy Cas9 proteins were expressed in *E. coli* DH10B while Blat Cas9 in *E. coli* BL21 (DE3) strains grown in LB broth supplemented with ampicillin (100 mg/mL). After growing bacteria at 37 °C and reaching an OD600 of 0.5, temperature was decreased to 16 °C and expression induced with 0.2 % (w/v) arabinose for 20 h. Cells were pelleted and re-suspended in loading buffer (20 mM KH_2_PO_4_ pH 7.0, 0.5 M NaCl, 10 mM imidazole, 5 % glycerol) and disrupted by sonication. Cell debris was removed by centrifugation. The supernatant was loaded onto the Ni^2+^-charged 5 mL HiTrap chelating HP column (GE Healthcare) and eluted with a linear gradient of increasing imidazole concentration. The fractions containing Cas9 were pooled and subsequently loaded onto HiTrap heparin HP column (GE Healthcare) for elution using a linear gradient of increasing NaCl concentration (from 0.5 to 1 M NaCl). The fractions containing Cas9 were pooled and dialyzed against 10 mM Bis-Tris–HCl, pH 7.0, 300 mM KCl, 1 mM EDTA, 1 mM DTT, and 50 % (v/v) glycerol and stored at −20 °C.

### Synthesis of RNAs

Origins of all RNA molecules used in this study are listed in Additional file 1: Table S2.

### Construction of a 5 bp randomized PAM library

Construction of the 5 bp randomized PAM plasmid DNA library was initiated with the synthesis of a single oligonucleotide, GG-821N, with hand-mixing used to create a random incorporation of nucleotides across the five random residues (represented as N in the sequence of GG-821N). To convert the single-stranded template of GG-821N into a double-stranded DNA template for cloning into the plasmid vector, a second oligonucleotide, GG-820, was synthesized with complementation to the 3′ end of GG-821N to form a partial oligonucleotide duplex. The partial duplex was then extended by PCR using DreamTaq polymerase (Thermo Fisher Scientific) to generate a full duplex containing the target sequence, five randomized base pairs downstream of the target sequence and cleavage site for the BamHI restriction enzyme. To generate the plasmid library, the oligoduplex, purified using GeneJET PCR Purification Kit (Thermo Fisher Scientific), was digested with BamHI and ligated into pTZ57R/T vector (Thermo Fisher Scientific) pre-cleaved with BamHI. Linear pTZ57R/T vector contains protruding ddT nucleotide at the 3′ ends, whereas PCR fragments generated with DreamTaq polymerase contains dA at the 3′ ends. Therefore one end of the PCR fragment is ligated into the vector through BamHI sticky ends, while another through A/T ends. DH5α Ca^2+^ competent cells were transformed with the ligated plasmid library and plated onto Luria Broth (LB) containing agar. The transformation efficiency was estimated from plated dilutions. Overall, approximately 12,000 colonies were recovered. The colonies were harvested from the plate by gently re-suspending them in liquid LB media and plasmid DNA was purified using GeneJET Plasmid Miniprep kit (Thermo Fisher Scientific).

### Construction of a 7 bp randomized PAM library

The 7 bp randomized PAM plasmid DNA library was constructed as described for the 5 bp library with the following modifications. Randomization of the PAM sequence was generated through the synthesis of four oligonucleotides, GG-940-G, GG-940-C, GG-940-A, and GG-940-T, with hand-mixing used to create a random incorporation of nucleotides across the random residues (represented as N). The randomized single-stranded oligonucleotides were each separately converted into double-stranded DNA templates for cloning into the plasmid vector using a second oligonucleotide, GG-939, with complementation to the 3′ end of GG-940-G, GG-940-C, GG-940-A, and GG-940-T and by PCR extension with DreamTaq polymerase (Thermo Fisher Scientific). To avoid cleavage of some species of the randomized positions, the resulting double-stranded templates were each digested with an 8 bp cutting restriction endonuclease, SdaI, so that overhangs were present at each end; a PstI compatible overhang and a Taq added single 3′ A overhang. The resulting overhangs were used to directionally ligate the four double-stranded templates into pTZ57R/T (Thermo Fisher Scientific) pre-cleaved with PstI. The ligations were transformed into DH5α Ca^2+^ competent cells, plasmid DNA was recovered and combined from each of the four transformants derived from GG-940-G, GG-940-C, GG-940-A, and GG-940-T to generate the randomized 7 bp PAM plasmid DNA library.

### PAM library validation

To validate the randomness of the resulting PAM library, PCR fragments spanning the 5 bp and 7 bp randomized PAM regions were generated by Phusion High-Fidelity DNA Polymerase (Thermo Fisher Scientific) amplification (15 cycles of a two-step amplification protocol) using the primer pair combinations TK-119/pUC-dir and TK-113/pUC-dir for the 5 bp and 7 bp libraries, respectively. The resulting 145 bp PCR product was purified using GeneJET PCR Purification Kit (Thermo Fisher Scientific) and the sequences necessary for amplicon-specific barcodes and Illumina sequencing were ‘tailed’ on through two rounds of PCR, each consisting of 10 cycles. The primer pair combinations in the first round of PCR were JKYS800.1/JKYS803 and JKYS921.1/JKYS812 for the 5 bp and 7 bp libraries, respectively. A set of primers, JKYS557/JKYS558, universal to all primary PCR reactions was utilized for the secondary PCR amplification. The resulting PCR amplifications were purified with a Qiagen PCR purification spin column, concentration measured with a Hoechst dye-based fluorometric assay, combined in an equimolar ratio, and single-read 60–100 nucleotide-length deep sequencing was performed on Illumina’s MiSeq Personal Sequencer with a 5–10 % (v/v) spike of PhiX control v3 (Illumina, FC-110-3001) to offset sequence bias. After sequencing, reads were trimmed to a minimum Phred quality (Q score) of 13 and different treatments were deconvoluted by identifying a perfectly matching 4–6 nt barcode sequence present at the 5 prime end. The PAM sequence for only those reads containing a perfect 12 nt sequence match flanking either side of the randomized PAM sequence were captured. The collection of resulting PAM sequences were then collapsed into like sequences, counted, and frequency of each PAM calculated. A position frequency matrix (PFM) was then performed by first aligning the collapsed PAM sequences. Next, each nucleotide (G, C, A, or T) at each position of the PAM was weighted based on the frequency of the PAM sequence with which it was associated. Finally, the total contribution of each nucleotide (G, C, A, or T) at each PAM position was summed to generate the overall probability of identifying a given nucleotide at each PAM position within the dataset (Additional file 1: Figure S2A and B).

### Assembly of Cas9 RNP complexes

Cas9-guide RNA complexes were assembled by mixing Cas9 protein with pre-annealed crRNA and tracrRNA duplex or sgRNA at 1:1 molar ratio followed by incubation in a complex assembly buffer (10 mM Tris–HCl pH 7.5 at 37 °C, 100 mM NaCl, 1 mM EDTA, 1 mM DTT) at 37 °C for 1 h.

### Digestion of plasmid libraries

One microgram (5.6 nM) of plasmid DNA library with randomized PAM was cleaved with 0.5 nM and 50 nM of Cas9-guide RNA complex in a reaction buffer (10 mM Tris–HCl pH 7.5 at 37 °C, 100 mM NaCl, 10 mM MgCl2, 1 mM DTT) for 60 min at 37 °C in a 100 μL reaction volume.

### Capture and identification of PAM preferences

To efficiently capture the blunt ends of the plasmid library generated by Cas9-guide RNA complex cleavage, a 3′ dA was added by incubating the completed digestion reactions with 2.5 U of DreamTaq DNA Polymerase (Thermo Fisher Scientific) and 0.5 μL of 10 mM dATP (or dNTP) for an additional 30 min. at 72 °C. Reaction products were purified using GeneJET PCR Purification Kit (Thermo Fisher Scientific). Next adapters with a 3′ dT overhang were generated by annealing TK-117 and phosphorylated TK-111 oligonucleotides. 100 ng of the resulting adapter was ligated to an equal concentration of the purified 3′ dA overhanging cleavage products for 1 h at 22 °C in a 25 μL reaction volume in ligation buffer (40 mM Tris–HCl pH 7.8 at 25 °C, 10 mM MgCl_2_, 10 mM DTT, 0.5 mM ATP, 5 % (w/v) PEG 4000, and 0.5 U T4 Ligase; Thermo Fisher Scientific). Next, to selectively enrich for cleaved products containing the PAM sequence, PCR amplification was performed with a forward primer, pUC-dir specific to the PAM-side of the cleaved pTZ57R/T plasmid vector and with a reverse primer, TK-117 specific to the ligated TK-117/TK-111 adapter sequence. PCR fragments were generated by Phusion High-Fidelity DNA Polymerase (Thermo Fisher Scientific) amplification (15 cycles of a two-step amplification protocol) with 10 μL of ligation reaction mixtures as a template (in 100 μL total volume). The resulting 131 bp PCR products amplified from the Cas9-guide RNA complex cleaved plasmid libraries were purified with GeneJET PCR Purification Kit (Thermo Fisher Scientific) and prepared for Illumina deep sequencing as described in the PAM library validation section except the barcode containing forward primers used in the primary reaction were specific to the TK-117/TK-111 adapter sequence. Illumina deep sequencing, post-processing, and position frequency matrices (PFMs) were performed as described in the PAM library validation section. WebLogos were generated as described by [23].

### Determination of the cleavage position in the protospacer

A total of 2.5 μg of pUC18 plasmid with cloned T1 spacer and GTCCCGAA PAM sequence was digested with 100 nM of the Blat Cas9-sgRNA complex in 500 μL of reaction buffer at 37 °C for 60 min., purified using GeneJET PCR Purification Kit (Thermo Fisher Scientific), and electrophoresed on an agarose gel. Linear digestion products were then purified from the agarose gel using the GeneJET Gel Extraction Kit (Thermo Fisher Scientific). To examine the exact cleavage position of the target sequence, the cleaved plasmid was directly sequenced with the pUC-EheD and pUC-LguR primers.

### PAM confirmation

For *in vitro* confirmation of the PAM preferences of Blat Cas9, cleavage reactions were initiated by mixing supercoiled plasmid DNA with pre-assembled Blat Cas9-sgRNA complex (1:1 v/v ratio) at 15 °C. The final reaction mixture contained 3 nM plasmid, 50 nM Cas9, 10 mM Tris–HCl (pH 7.5 at 37 °C), 100 mM NaCl, 1 mM DTT, and 10 mM MgCl_2_ in a 100 μL reaction volume. Aliquots were removed at timed intervals and quenched with phenol/chloroform. The aqueous phase was mixed with 3× loading dye solution (0.01 % (w/v) bromophenol blue and 75 mM EDTA in 50 % (v/v) glycerol) and reaction products analyzed by agarose gel electrophoresis. The amount of supercoiled (SC) form was evaluated by densitometric analysis of ethidium bromide stained gels using the software ImageJ. Values of reaction rate constants were obtained as described earlier [8].

### *In planta* mutation detection

The DNA region surrounding the expected site of cleavage for each Cas9-guide RNA was amplified by PCR using Phusion® High Fidelity PCR Master Mix (NEB, USA) ‘tailing’ on the sequences necessary for amplicon-specific barcodes and Illumina sequences through two rounds of PCR each consisting of 20 cycles. The primer pairs used in the primary PCR were JKYX1.1/JKYS178Rd, JKYS1083.1/JKYS1084, and JKYX2.1/JKYX3 each corresponding to *Ms45* exon 1, *Ms45* exon 4, and *liguleless-1* targets, respectively. A set of primers universal to the products from the primary reactions, JKY557/JKY558, were used in the secondary PCR reaction. The resulting PCR amplifications were purified with a Qiagen PCR purification spin column (Qiagen, Germany), concentration measured with a Hoechst dye-based fluorometric assay, combined in an equimolar ratio, and single-read 100 nucleotide-length amplicon sequencing was performed on Illumina’s MiSeq Personal Sequencer with a 5–10 % (v/v) spike of PhiX control v3 (Illumina, FC-110-3001) to offset sequence bias. Post-processing on the resulting sequences was performed as described in the PAM library validation section and only those reads with a ≥1 nucleotide INDEL arising within the 10 nt window centered over the expected site of cleavage and not found in the negative controls were classified as mutations. Mutant reads with an identical mutation were counted and collapsed into a single read and the top 10 most prevalent mutations were visually confirmed as arising within the expected site of cleavage. The total numbers of visually confirmed mutations were then used to calculate the percentage of mutant reads based on the total number of reads of an appropriate length containing a perfect match to the barcode and forward primer.

### Data availability

Raw deep sequencing data are available at the NCBI archive under Bioproject Accession number PRJNA299513.

## Additional file

Additional file 1:
**Figures S1–S14 and Tables S1 and S2.** (PDF 993 kb)

## Abbreviations

Blat: *Brevibacillus laterosporus*
bp: base pair
cas: CRISPR-associated
CRISPR: clustered regularly interspaced short palindromic repeats
crRNA: CRISPR RNA
dA: 3′-deoxyadenosine
ddT: 2′, 3′- dideoxythymidine
dT: 3′-deoxythymidine
DTT: dithiothreitol
EDTA: ethylenediaminetetraacetic acid
IMEs: immature maize embryos
PAM: protospacer adjacent motif
PCR: polymerase chain reaction
sgRNA: single-guide RNA
Spy: *Streptococcus pyogenes*
Sth1: *Streptococcus thermophilus* CRISPR1
Sth3: *Streptococcus thermophilus* CRISPR3
tracrRNA: trans activating CRISPR RNA
Tris: tris(hydroxymethyl)aminomethane
