# Supplementary material for: Rapid characterization of CRISPR-Cas9 protospacer adjacent motif sequence elements
Source: Genome Biol. 2015 Nov 19;16:253. doi: 10.1186/s13059-015-0818-7 (PMC4653880; doi:10.1186/s13059-015-0818-7)
Supplement: Additional file 1: — Figures S1–S14 and Tables S1 and S2. (PDF 993 kb) [file 13059_2015_818_MOESM1_ESM.pdf]

Supporting Information

Rapid Characterization of Cas9 Protospacer Adjacent Motif Sequence Elements

Tautvydas Karvelis, Giedrius Gasiunas, Joshua Young, Greta Bigelyte, Arunas Silanskas, Mark Cigan and Virginijus Siksnys

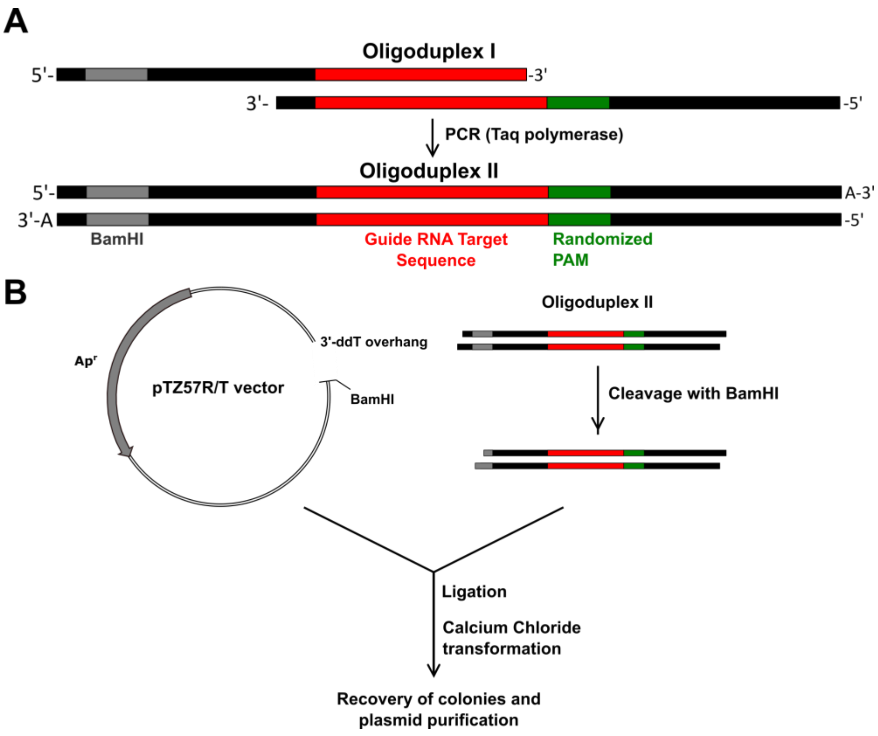

Supplementary Figures S1A & B. Schematic for generating randomized PAM plasmid DNA libraries. A. Single-stranded oligonucleotide containing the randomized PAM region is hybridized to a second oligonucleotide forming oligoduplex I and converted into a fully double-stranded DNA, oligoduplex II, by extension with Taq polymerase.The region containing the randomized PAM sequence is in green and the guide RNA target sequence region is in red. **B.** Oligoduplex II is cloned into the plasmid DNA vector over BamHI and 3' dA overhanging ends, transformed into *E. coli* and plasmid vector DNA recovered.

A

		PAM Position 1	2	3	4	5
Nucleotide	G	22.88%	23.06%	26.94%	23.81%	25.63%
	C	28.07%	26.92%	27.55%	28.34%	26.71%
	A	25.86%	26.89%	25.29%	24.90%	25.77%
	T	23.18%	23.13%	20.23%	22.96%	21.89%
	Average	25.00%	25.00%	25.00%	25.00%	25.00%
	S.E.M.	1.22%	1.10%	1.66%	1.18%	1.06%

B

		PAM Position 1	2	3	4	5	6	7
Nucleotide	G	23.33%	20.49%	21.49%	20.41%	22.41%	22.38%	20.83%
	C	35.35%	29.44%	30.95%	30.33%	27.56%	30.33%	31.78%
	A	19.30%	31.08%	28.83%	29.95%	26.46%	28.67%	26.70%
	T	22.02%	18.99%	18.73%	19.30%	23.57%	18.62%	20.68%
	Average	25.00%	25.00%	25.00%	25.00%	25.00%	25.00%	25.00%
	S.E.M.	3.55%	3.07%	2.91%	2.98%	1.20%	2.73%	2.66%

Supplementary Figures S2A & B. Nucleotide composition of the 5 bp (A) and 7 bp (B) randomized PAM regions. Position frequency matrix (PFM) [1] was used to calculate the nucleotide composition at each position of the PAM region in the 5 bp and 7 bp randomized PAM libraries to ensure a near random and balanced incorporation of each base pair.

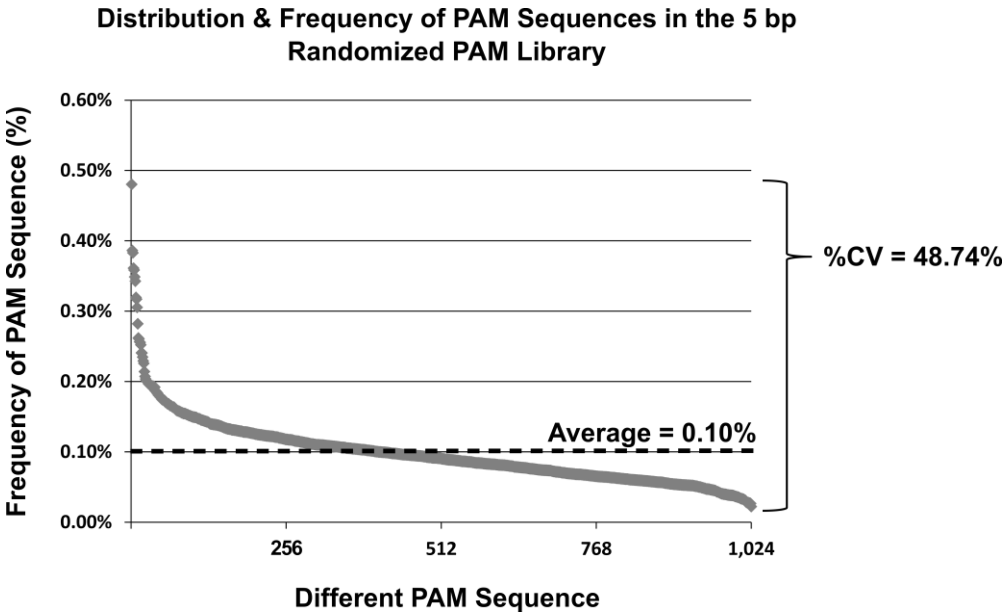

Supplementary Figure S3. PAM sequence distribution from a 5 nucleotide (5N) randomized Protospacer Adjacent Motif (PAM) plasmid library. The PAM sequence distribution was visualized by ordering the frequency of each PAM from greatest to least and displaying them graphically. All 1,024 possible PAM sequences were present at an average frequency of 0.10% with a coefficient of variation (CV) of 48.74%.

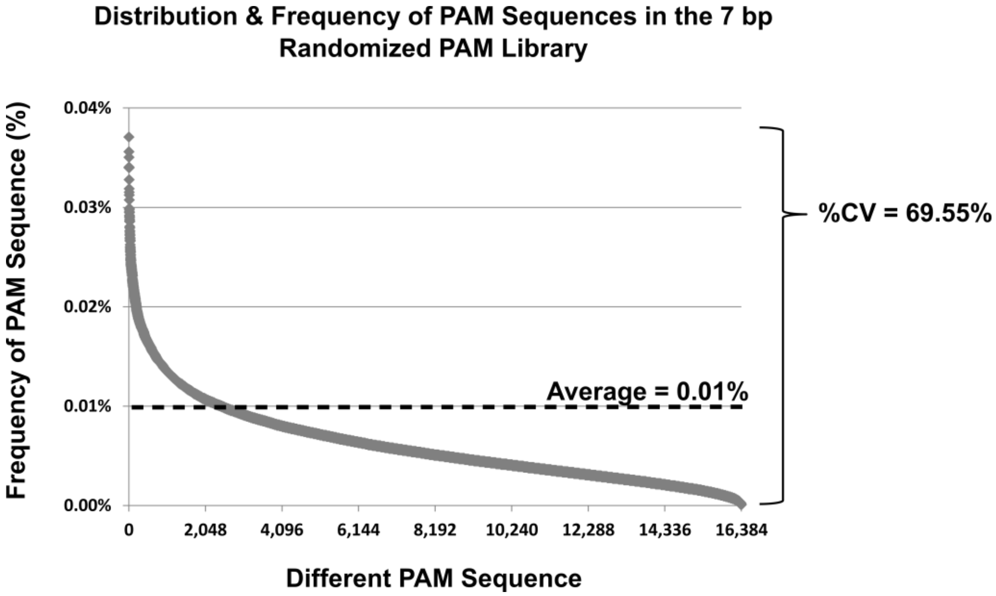

Supplementary Figure S4. PAM sequence distribution from a 7 nucleotide (7N) randomized Protospacer Adjacent Motif (PAM) plasmid library. The PAM sequence distribution was visualized by ordering the frequency of each PAM from greatest to least and displaying them graphically. All 16,384 possible PAM sequences were present at an average frequency of 0.01% with a coefficient of variation (CV) of 69.55%.

A C

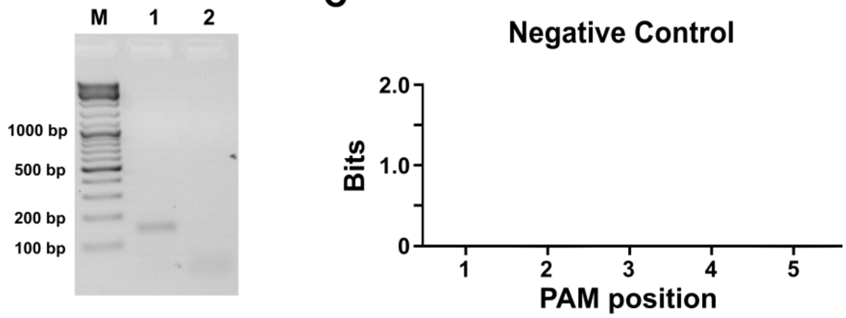

B

		PAM Position				
		1	2	3	4	5
Nucleotide	G	24.85%	23.46%	23.63%	23.81%	23.99%
	C	24.59%	24.51%	22.98%	23.91%	23.61%
	A	25.33%	25.75%	26.88%	25.14%	27.00%
	T	25.24%	26.28%	26.51%	27.14%	25.40%
	Average	25.00%	25.00%	25.00%	25.00%	25.00%
	S.E.M.	0.17%	0.63%	0.99%	0.78%	0.77%

Supplementary Figures S5A, B and C. PCR enrichment and analysis of plasmid DNA library in the absense of Cas9-guide RNA complexes. A. PCR products were only obtained with primer pair TK-119/pUC-dir designed to amplify uncut species of the randomized PAM library (lane 1) while primer pair TK-117/pUC-dir designed to amplify cleaved species of the randomized PAM library to which an adapter had been ligated failed to yield PCR amplification products (lane 2). **B & C.** Analysis of nucleotide distribution in the randomized PAM region of a PCR product (generated using primer pair TK-119/pUC-dir) obtained from the 5 bp randomized PAM library in the absence of Cas9-guide RNA complex digestion. Data is presented in two forms, as a position frequency matrix (PFM) ([1]) (**B**) and as a WebLogo [2] (**C**).

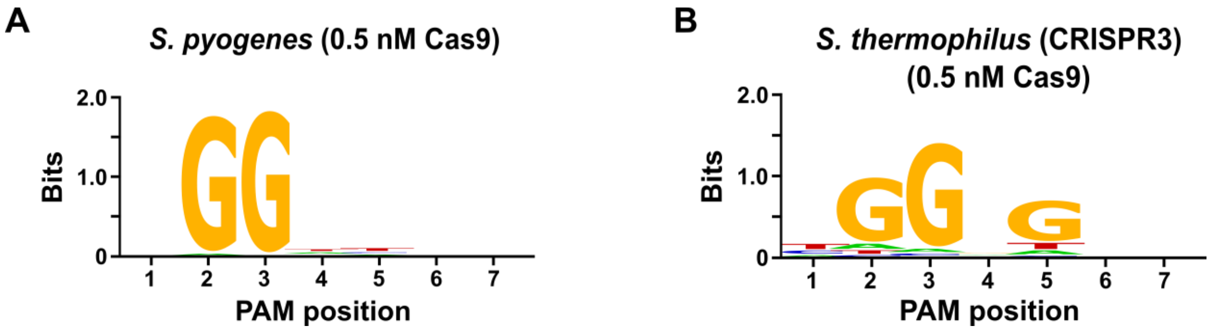

Supplementary Figures S6A and B. PAM preferences for *S. pyogenes* (A) and *S. thermophilus* CRISPR3 (B) Cas9 proteins with the 7 bp randomized PAM library. Frequency of nucleotides at each PAM position was independently calculated using a position frequency matrix (PFM) [1] and plotted as a WebLogo [2].

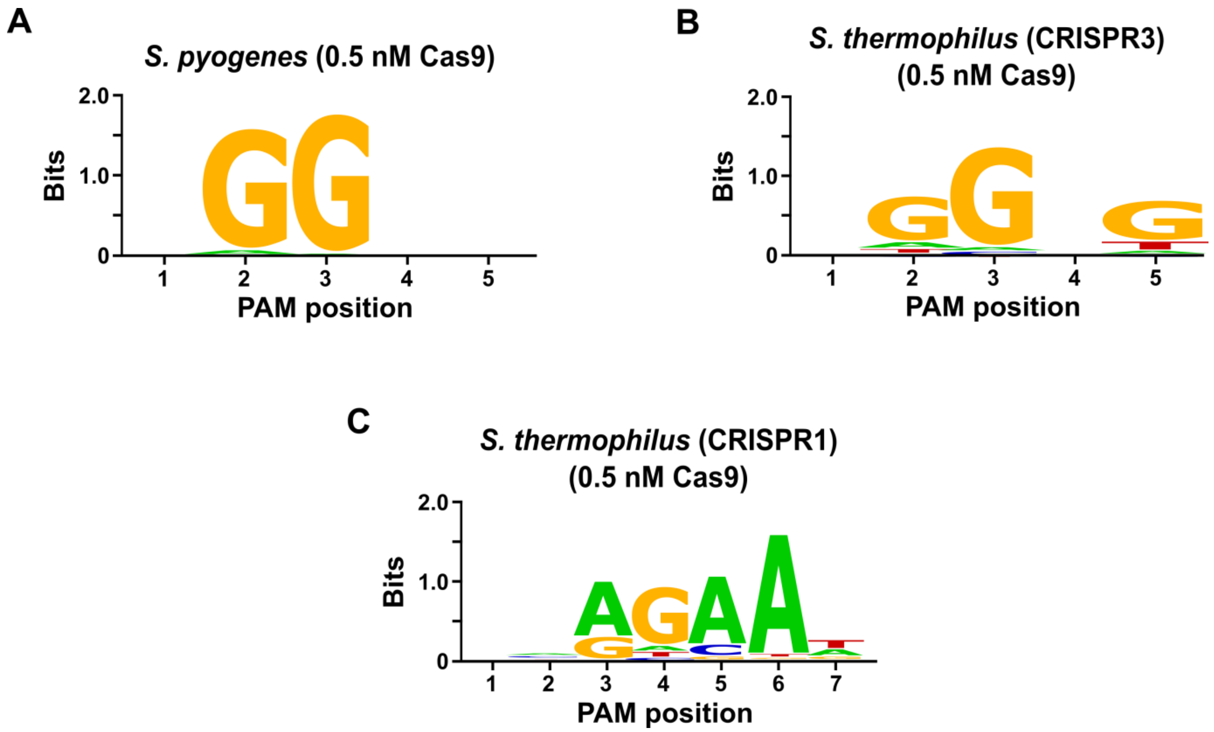

Supplementary Figures S7A, B and C. PAM preferences for *S. pyogenes* (A), *S. thermophilus* CRISPR3 (B) and *S. thermophilus* CRISPR1 (C) Cas9 proteins when complexed with a single guide RNA (sgRNA). Frequency of nucleotides at each PAM position was independently calculated using a position frequency matrix (PFM) [1] and plotted as a WebLogo [2].

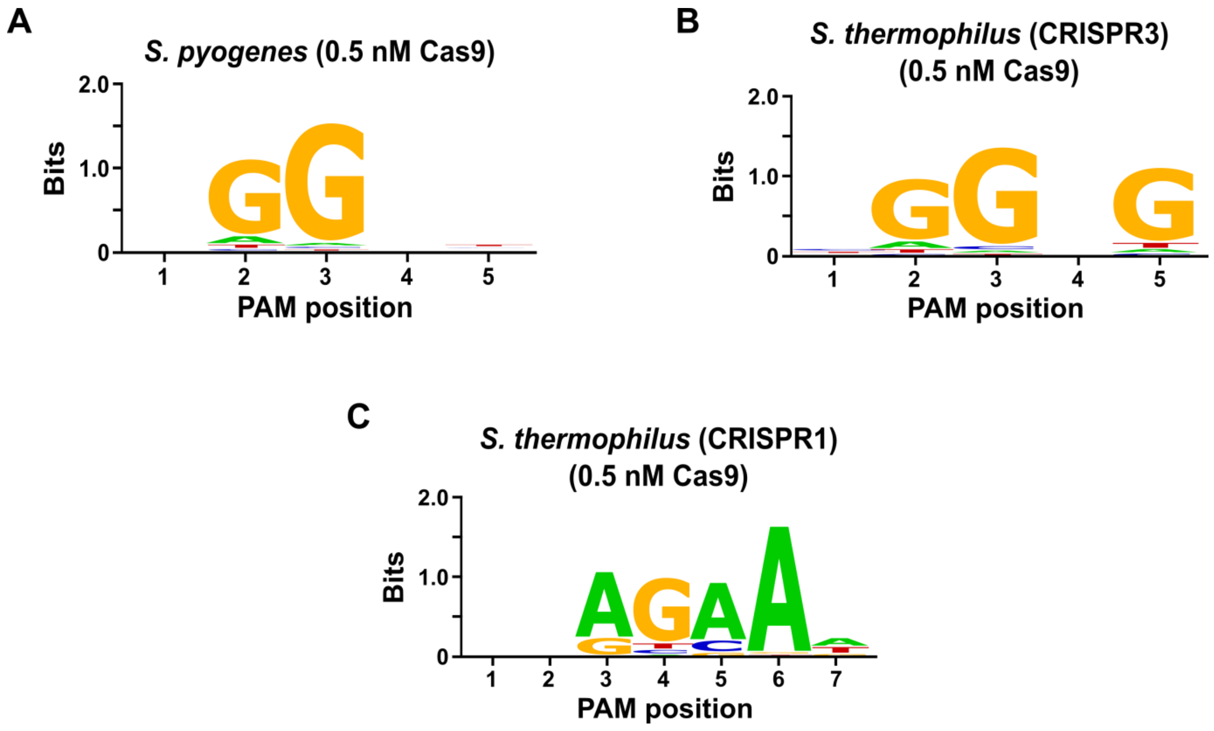

Supplementary Figures S8A, B and C. PAM preferences for *S. pyogenes* (A), *S. thermophilus* CRISPR3 (B) and *S. thermophilus* CRISPR1 (C) Cas9 proteins using a different spacer and protospacer target. Frequency of nucleotides at each PAM position was independently calculated using a position frequency matrix (PFM) [1] and plotted as a WebLogo [2].

ATCATATCATATCGAGTTTTAGTAAGGAACTATAGCACCTGCCGCTAAGACCACTAAATCTGATACATCATATCATATCGAG
CTTTAGTAAGGAACTATAGCTTGGACCTACTATGGCATATATACATTTAGATCATATCATATCGAGTTTTAGTAAGGAACCA
TAGCCTACAATAACAATTTCCCTCATACCACACTATCATATCATATCGAGTTTTAGTAAGGAACTATAGCCCAATTAAAGAA
GGTATCACAGATTGCTTTATCATATCATATCGAGTTTTAGTAAGGAACTATAGCAACAGTCGTATCGTTGTATTTATCAATA
GATCATATCATATCGAGCTTCAGTAAGGAACTATAGCGACATTGGTTGCGGCAATAAGACAGTACGCATCATATCATATCAA
GCTTTAGTAAGGAACTATAGCAGCAATATGCTAGCCCTCACATTTACCCGAATCATATCATATCGAGTTTTAGTAAGGAACT
ATAGT

Supplementary Figure S9. CRISPR array repeats of the Type II CRISPR-Cas system from *B. laterosporus* SSP360D4. Repeats are shown in blue with mismatches shown in red. Spacer sequences are shown in black.

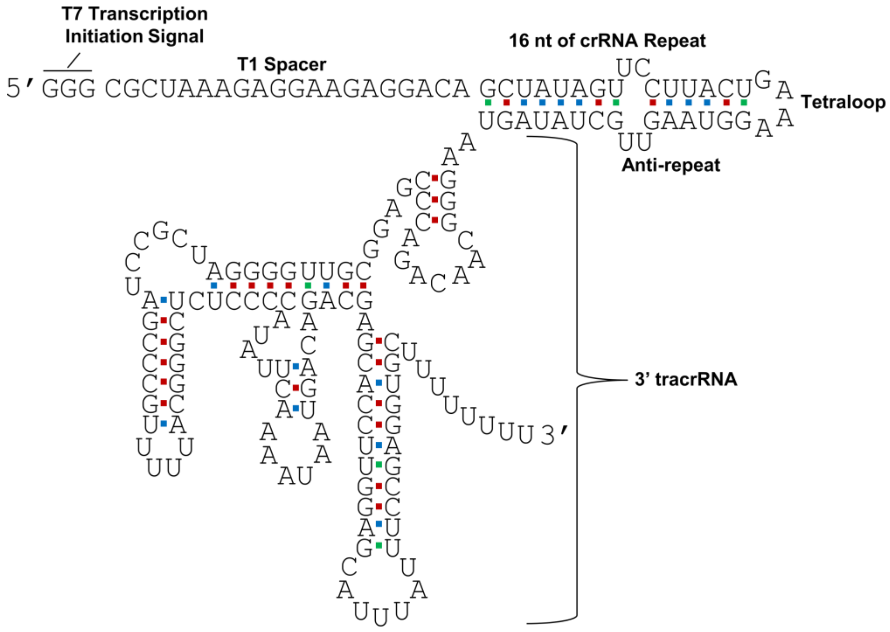

Supplementary Figure S10. Schematic of the "direct" sgRNA supporting *in vitro* cleavage for *B. laterosporus* SSP360D4 Cas9. Secondary structures were predicted using UNAfold [3].

		PAM Position						
		1	2	3	4	5	6	7
Nucleotide	G	20.87%	18.43%	19.33%	12.94%	0.27%	25.40%	20.87%
	C	32.72%	27.42%	25.45%	40.59%	96.53%	30.72%	0.69%
	A	20.66%	24.24%	28.25%	10.74%	2.86%	36.17%	69.51%
	T	25.75%	29.90%	26.97%	35.74%	0.34%	7.71%	8.93%

Supplementary Figure S11. Position frequency matrix (PFM) of *B. laterosporus* SSP360D4 Cas9 cleaved PAM sequences. Position frequency matrix (PFM) [1] of the PAM sequences supporting cleavage under the 0.5 nM Blat Cas9 "direct" sgRNA RNP complex digest conditions in the 7 bp randomized PAM library.

ATGGCCCCCAAGAAGAAAAGGAAGGTCATGGCCTACACGATGGGCATCGACGTCGGCATCGCCAGCTGCGGCTGGGCCATCGTCGA
CCTGGAGAGGCAGCGAATAATAGACATCGGCGTCCGCACCTTCGAGAAGGTAAGTTTCTGCTTCTACCTTTGATATATATATAATA
ATTATCATTAATTAGTAGTAATATAATATTTCAAATATTTTTTTCAAAATAAAAGAATGTAGTATATAGCAATTGCTTTTCTGTAG
TTTATAAGTGTGTATATTTTAATTTATAACTTTTCTAATATATGACCAAAACATGGTGATGTGCAGGCCGAGAACCCCAAGAACGG
CGAGGCCCTCGCCGTCCCGAGGAGGGAGGCCAGGAGCTCGAGGAGGAGGCTGAGGAGGAAGAAGCACCGCATCGAGAGATTAAAGC
ATATGTTCGTCCGCAACGGCCTCGCCGTCGACATCCAGCACCTCGAGCAGACCCTCCGCTCCCAGAATGAAATAGACGTCTGGCAG
CTCCGCGTCGACGGCCTCGACAGGATGCTGACCCAGAAAGAGTGGCTGCGCGTATTAATTCACCTCGCCCAGCGCCGCGGCTTCCA
ATCAAATCGCAAGACCGACGGCAGCAGCGAGGACGGCCAGGTCCTCGTCAACGTCACCGAGAACGACAGATTAATGGAGGAGAAGG
ACTACAGGACCGTCGCCGAGATGATGGTGAAGGATGAAAAATTCAGCGACCACAAGCGCAATAAAAATGGCAACTACCACGGCGTC
GTCAGCCGCAGCAGCCTCCTCGTCGAAATACATACATTATTCGAGACCCAGAGGCAGCACCACAACAGCCTCGCCAGCAAGGACTT
CGAGCTCGAGTACGTCAACATCTGGAGCGCCCAGAGGCCCGTGGCCACGAAGGACCAGATCGAGAAGATGATCGGCACCTGCACCT
TCCTCCCCAAAGAGAAGCGCGCCCCCAAGGCCTCCTGGCACTTCCAGTACTTCATGCTCCTCCAGACCATCAACCACATCCGCATC
ACCAACGTCCAGGGCACCAGGAGCCTCAATAAAGAGGAGATCGAGCAGGTCGTCAATATGGCCCTCACCAAATCAAAGGTCAGCTA
CCACGATACTAGGAAGATCCTCGACCTCAGCGAGGAGTACCAGTTCGTCGGCCTCGACTACGGCAAGGAGGATGAAAAAAAAAAGG
TGGAGAGCAAGGAGACGATCATCAAGCTCGACGACTACCACAAGCTCAACAAGATCTTCAACGAGGTGGAGCTGGCGAAAGGGGAG
ACGTGGGAGGCCGACGACTACGACACCGTCGCCTACGCCCTCACCTTCTTCAAGGACGACGAGGACATCCGCGACTACCTCCAGAA
TAAATATAAAGACAGCAAAAACCGGCTGGTGAAGAACCTCGCCAATAAAGAATACACAAACGAGCTCATCGGCAAGGTCAGCACCC
TCAGCTTCAGGAAGGTCGGCCACCTCAGCCTCAAGGCCCTCAGGAAGATCATCCCCTTCCTCGAGCAGGGCATGACCTACGACAAG
GCCTGCCAGGCCGCCGGCTTCGACTTCCAGGGCATCAGCAAGAAGAAGAGGAGCGTGGTCCTCCCCGTCATCGACCAGATCAGCAA
CCCCGTCGTCAACAGGGCCCTCACCCAGACCAGGAAGGTAATTAATGCCCTAATCAAAAAATATGGCAGCCCCGAGACCATCCACA
TCGAGACGGCGAGGGAGCTGTCGAAAACGTTCGACGAGAGGAAGAACATCACCAAAGACTACAAGGAGAACCGGGACAAGAACGAG
CACGCGAAGAAACACCTCAGCGAGCTCGGCATCATTAATCCGACGGGCCTGGACATCGTGAAATACAAGCTCTGGTGCGAGCAGCA
GGGCAGGTGCATGTACAGCAATCAACCCATCAGCTTCGAGCGCCTCAAGGAGAGCGGCTACACCGAGGTCGACCACATCATCCCCT
ACAGCCGCAGCATGAACGACAGCTACAACAACCGCGTCCTCGTCATGACCCGCGAGAACCGCGAGAAGGGCAATCAAACCCCCTTC
GAGTACATGGGCAACGACACCCAGCGCTGGTACGAGTTCGAGCAGCGCGTCACCACCAACCCGCAAATAAAAAAGGAGAAGCGCCA
GAACCTCCTCCTCAAGGGCTTCACCAACCGCAGGGAGCTCGAGATGCTCGAGAGGAACCTCAACGACACCCGCTATATAACTAAGT
ACCTCAGCCACTTCATCAGCACCAACCTCGAGTTCAGCCCCAGCGATAAAAAGAAGAAGGTCGTCAATACATCTGGGAGGATCACC
AGCCACCTCAGGAGCCGCTGGGGCCTGGAGAAGAACCGCGGCCAGAACGACCTCCACCACGCGATGGACGCCATCGTCATCGCCGT
CACCAGCGACTCCTTCATCCAGCAGGTCACCAACTACTACAAGAGGAAGGAGCGCCGCGAGCTCAACGGCGACGACAAGTTCCCCC
TCCCCTGGAAGTTCTTCCGCGAGGAGGTCATCGCCCGCCTCAGCCCGAACCCCAAGGAGCAGATCGAAGCATTGCCCAACCACTTC
TACAGCGAGGACGAGCTCGCCGACCTCCAGCCCATCTTCGTCAGCCGCATGCCCAAGCGCAGCATCACCGGCGAGGCCCACCAGGC
CCAGTTCCGCCGCGTCGTCGGCAAGACCAAGGAGGGCAAAAATATTACCGCCAAAAAAACCGCCCTCGTCGACATCAGCTACGATA
AAAATGGCGACTTCAACATGTACGGCCGCGAGACCGACCCCGCCACCTACGAGGCAATTAAAGAGCGCTACCTCGAGTTCGGCGGC
AACGTCAAGAAAGCATTTAGCACCGACCTCCATAAACCCAAAAAGGACGGCACCAAGGGGCCCCTCATAAAAAGCGTCAGAATAAT
GGAAAATAAAACACTCGTCCACCCCGTCAATAAAGGCAAGGGCGTCGTCTACAACAGCAGCATCGTCCGCACCGACGTGTTCCAGC
GCAAGGAGAAGTACTACCTCCTCCCCGTCTACGTCACCGACGTAACCAAGGGCAAGCTCCCCAACAAGGTCATCGTCGCCAAGAAG
GGCTACCACGACTGGATCGAGGTCGACGACAGCTTCACCTTCCTCTTCAGCCTCTACCCCAACGACCTCATCTTCATCAGGCAGAA
CCCCAAGAAGAAAATATCATTAAAAAAGCGCATCGAGAGCCACAGCATCAGCGACAGCAAGGAGGTCCAGGAGATCCACGCCTACT
ACAAGGGCGTCGACAGCAGCACCGCCGCCATCGAGTTCATCATCCACGACGGCAGCTACTACGCCAAGGGCGTCGGCGTCCAGAAC
CTCGACTGCTTCGAAAAATATCAGGTCGACATCCTCGGCAACTACTTCAAGGTCAAGGGCGAGAAGAGGCTCGAGCTCGAGACCAG
CGACAGCAACCACAAGGGCAAGGACGTCAACAGCATTAAATCAACTAGCAGGAAGAGGCCCAGGGACCGCCACGACGGCGAGCTCG
GCGGCAGGAAGAGGGCCCGC

Supplementary Figure S12. Maize optimized *B. laterosporus* SSP360D4 *cas9* gene. Open reading frame of the *cas9* gene, amino and carboxyl-terminal nuclear localization signals (blue highlighted text) and Intron 2 of the potato *ST-LSI* gene (yellow highlighted text) are shown.

TGAGAGTACAATGATGAACCTAGATTAATCAATGCCAAAGTCTGAAAAATGCACCCTCAGTCTATGATCCAGAAAATCAAGATTGC
TTGAGGCCCTGTTCGGTTGTTCCGGATTAGAGCCCCGGATTAATTCCTAGCCGGATTACTTCTCTAATTTATATAGATTTTGATGA
GCTGGAATGAATCCTGGCTTATTCCGGTACAACCGAACAGGCCCTGAAGGATACCAGTAATCGCTGAGCTAAATTGGCATGCTGTC
AGAGTGTCAGTATTGCAGCAAGGTAGTGAGATAACCGGCATCATGGTGCCAGTTTGATGGCACCATTAGGGTTAGAGATGGTGGCC
ATGGGCGCATGTCCTGGCCAACTTTGTATGATATATGGCAGGGTGAATAGGAAAGTAAAATTGTATTGTAAAAAGGATTTCTTCT
GTTTGTTAGCGCATGTACAAGGAATGCAAGTTTTGAGCGAGGGGGCATCAAAGATCTGGCTGTGTTTCCAGCTGTTTTTGTTAGCC
CCATCGAATCCTTGACATAATGATCCCGCTTAAATAAGCAACCTCGCTTGTATAGTTCCTTGTGCTCTAACACACGATGATGATAA
GTCGTAAAATAGTGGTGTCCAAAGAATTTCCAGGCCCAGTTGTAAAAGCTAAAATGCTATTCGAATTTCTACTAGCAGTAAGTCGT
GTTTAGAAATTATTTTTTTATATACCTTTTTTCCTTCTATGTACAGTAGGACACAGTGTCAGCGCCGCGTTGACGGAGAATATTTG
CAAAAAAGTAAAAGAGAAAGTCATAGCGGCGTATGTGCCAAAAACTTCGTCACAGAGAGGGCCATAAGAAACATGGCCCACGGCCC
AATACGAAGCACCGCGACGAAGCCCAAACAGCAGTCCGTAGGTGGAGCAAAGCGCTGGGTAATACGCAAACGTTTTGTCCCACCTT
GACTAATCACAAGAGTGGAGCGTACCTTATAAACCGAGCCGCAAGCACCGAATTGATAATGAGGTACTGGCTGGAGCTATAGTTCC
TTACTGAAAGGTAAGTTGCTATAGTAAGGGCAACAGACCCGAGGCGTTGGGGATCGCCTAGCCCGTGTTTACGGCTCTCCCCATA
TTCAAAATAATGACAGACGAGCACCTTGGAGCATTTATCTCCGAGGTGCTTTTTTTTT

Supplementary Figure S13. Maize optimized *B. laterosporus* SSP360D4 single guide RNA (sgRNA) expression cassette. Maize U6 polymerase III promoter region is shown in black font. The region encoding the spacer targeting exon 4 of the maize fertility gene *Ms45* gene is blue highlighted black font. The repeat, self-folding tetraloop, anti-repeat and 3’ tracrRNA region are indicated in yellow highlighted black font while modifications to remove putative sites of transcriptional termination are underlined. The maize U6 polymerase III terminator is represented by red font.

	Target ↓ PAM	Frequency in Treated	Frequency in Control
**Reference**	**CCAGGATAATGAGGTACTGGCTGGAAGGCCCAAGAGCGGGCGAGGT**		
Mutation 1	CAGGATAATGAGGTACTGGCTtGGAAGGCCCAAGAGCGGGCGAGGT	2,807	0
Mutation 2	CCAGGATAATGAGGTACTGGC-GGAAGGCCCAAGAGCGGGCGAGGT	579	2
Mutation 3	CCAGGATAATGAGGTAC----TGGAAGGCCCAAGAGCGGGCGAGGT	130	0
Mutation 4	CCAGGATAATGAGGTACTGG-TGGAAGGCCCAAGAGCGGGCGAGGT	92	0
Mutation 5	CAGGATAATGAGGTACTGGCgTGGAAGGCCCAAGAGCGGGCGAGGT	85	0
Mutation 6	CCAGGATAATGAGGTACT--------------------GGCGAGGT	63	0
Mutation 7	CCAGGATAAT-------------------------------GAGGT	60	0
Mutation 8	CCAGGATAATGAGGTAC-----GGAAGGCCCAAGAGCGGGCGAGGT	48	0
Mutation 9	CAGGATAATGAGGTACTGGCTaGGAAGGCCCAAGAGCGGGCGAGGT	34	0
Mutation 10	CCAGGATAATGAGGTA------GGAAGGCCCAAGAGCGGGCGAGGT	33	0
	Total Read Depth	446,634	828,936

Supplementary Figure S14. ***S. pyogenes*** **Cas9 generates NHEJ mutations in maize.** Top 10 most prevalent types of NHEJ mutations detected with *S. pyogenes* Cas9 in exon 4 of the *Ms45* gene. Green font represents the spacer, while the blue font represents the overlapping PAM sequence of Spy (AGG) and Blat (AGGCCCCAA). A black arrow indicates the expected site of cleavage; mutations detected with Spy Cas9 are highlighted in red; lower case font indicates an insertion; "-" indicates a deletion.

Supplementary Table S1. Oligonucleotides and primers used in this study.

Oligonucleotide Name	Sequence (5' to 3')
Sth3-dir	GGGGGGTCTCACATGAGTGACTTAGT
Sth3-rev	AATTACTCGAGAAAATCTAGCTTAGGCTTA
Sth1-dir	ACGTCTCACATGACTAAGCCATACTCAATTGGAC
Sth1-rev	ACTCGAGACCCTCTCCTAGTTTGGCAA
Blat-dir	TACCATGGCATACACAATGGGAATAGATG
Blat-rev	TTCTCGAGACGACTAGTTGATTTAATCGAATTGAC
Spy-dir	AAGGTCTCCCATGGATAAGAAATACTCAATAGGCTTAG
Spy-rev	TTCTCGAGGTCACCTCCTAGCTGACTCAAATC
GG-821N	TGACCATGATTACGAATTCNNNNNTGTCCTCTTCCTCTTTAGCGAGC
GG-820	AAGGATCCCCGGGTACCGAGCTGCTCGCTAAAGAGGAAGAGGAC
GG-940-G	GTGCACGCCGGCGACGTTGGGTCAACTNNGNNNNTGTCCTCTTCCTCTTTAGCGTTTAG
GG-940-C	GTGCACGCCGGCGACGTTGGGTCAACTNNCNNNNTGTCCTCTTCCTCTTTAGCGTTTAG
GG-940-A	GTGCACGCCGGCGACGTTGGGTCAACTNNANNNNTGTCCTCTTCCTCTTTAGCGTTTAG
GG-940-T	GTGCACGCCGGCGACGTTGGGTCAACTNNTNNNNTGTCCTCTTCCTCTTTAGCGTTTAG
GG-939	GACTAGACCTGCAGGGGATCCCGTCGACAAATTCTAAACGCTAAAGAGGAAGAGGAC
TK-119	GAGCTCGCTAAAGAGGAAGAGG
pUC-dir	GCCAGGGTTTTCCCAGTCACGA
TK-113	GAAATTCTAAACGCTAAAGAGGAAGAGG
JKYS800.1	CTACACTCTTTCCCTACACGACGCTCTTCCGATCTAAGTGAGCTCGCTAAAGAGGAAGA
JKYS803	CAAGCAGAAGACGGCATACGAGCTCTTCCGATCTGAATTCGAGCTCGGTACCT
JKYS921.1	CTACACTCTTTCCCTACACGACGCTCTTCCGATCTGGAATAAACGCTAAAGAGGAAGAGG
JKYS812	CAAGCAGAAGACGGCATACGAGCTCTTCCGATCTCGGCGACGTTGGGTC
JKY557	AATGATACGGCGACCACCGAGATCTACACTCTTTCCCTACACG
JKY558	CAAGCAGAAGACGGCATA
TK-117	CGGCATTCCTGCTGAACCGCTCTTCCGATCT
TK-111	GATCGGAAGAGCGGTTCAGCAGGAATGCCG
pUC-EheD	CCGCATCAGGCGCCATTCGCC
pUC-LguR	GCGAGGAAGCGGAAGAGCGCCC
JKYX1.1	CTACACTCTTTCCCTACACGACGCTCTTCCGATCTAAGGGGCGCTGGCCCTCCTAGTC
JKYS178Rd	CAAGCAGAAGACGGCATACGAGCTCTTCCGATCTGCCGGCTGGCATTGTCTCTG
JKYS1083.1	CTACACTCTTTCCCTACACGACGCTCTTCCGATCTGGAAGGCAGGTTCGCGAACACCT
JKYS1084	CAAGCAGAAGACGGCATACGAGCTCTTCCGATCTCTCCGAGACAACAAACTGCAGGT
JKYX2.1	CTACACTCTTTCCCTACACGACGCTCTTCCGATCTAAGGGGCCGGACGCGGTGTT
JKYX3	CAAGCAGAAGACGGCATACGAGCTCTTCCGATCTTACATGCGCAGGTGCAAAGTCTAC

Supplementary Table S2. RNAs used in this study.

Name	Sequence (5’-3’)	Origin
Spy crRNA (T1)	CGCUAAAGAGGAAGAGGACAGUUUUAGAGCUAUGCUGUUUUG	Synthetic oligonucleotide
Spy tracrRNA	GGGAAACAGCAUAGCAAGUUAAAAUAAGGCUAGUCCGUUAUCAACU<br>UGAAAAAGUGGCACCGAGUCGGUGCUUUUUUU	*In vitro* transcription
Spy sgRNA (T1)	GGGCGCUAAAGAGGAAGAGGACAGUUUUAGAGCUAGAAAUAGCAAG<br>UUAAAAUAAGGCUAGUCCGUUAUCAACUUGAAAAAGUGGCACCGAG<br>UCGGUGCUUUUUU	*In vitro* transcription
Spy sgRNA (T2)	GGGUCUAGAUAGAUUACGAAUUCGUUUUAGAGCUAGAAAUAGCAAG<br>UUAAAAUAAGGCUAGUCCGUUAUCAACUUGAAAAAGUGGCACCGAG<br>UCGGUGCUUUUUU	*In vitro* transcription
Sth3 crRNA (T1)	CGCUAAAGAGGAAGAGGACAGUUUUAGAGCUGUGUUGUUUCG	Synthetic oligonucleotide
Sth3 tracrRNA	GGGCGAAACAACACAGCGAGUUAAAAUAAGGCUUAGUCCGUACUCA<br>ACUUGAAAAGGUGGCACCGAUUCGGUGUUUUU	*In vitro* transcription
Sth3 sgRNA (T1)	GGGCGCUAAAGAGGAAGAGGACAGUUUUAGAGCUGUGUUGUUUCGG<br>UUAAAACAACACAGCGAGUUAAAAUAAGGCUUAGUCCGUACUCAAC<br>UUGAAAAGGUGGCACCGAUUCGGUGUUUUUU	*In vitro* transcription
Sth3 sgRNA (T2)	GGGUCUAGAUAGAUUACGAAUUCGUUUUAGAGCUGUGUUGUUUCGG<br>UUAAAACAACACAGCGAGUUAAAAUAAGGCUUAGUCCGUACUCAAC<br>UUGAAAAGGUGGCACCGAUUCGGUGUUUUUU	*In vitro* transcription
Sth1 crRNA (T1)	CGCUAAAGAGGAAGAGGACAGUUUUUGUACUCUCAAGAUUUA	Synthetic oligonucleotide
Sth1 tracrRNA	GGGUAAAUCUUGCAGAAGCUACAAAGAUAAGGCUUCAUGCCGAAAU<br>CAACACCCUGUCAUUUUAUGGCAGGGUGUUUUCG	*In vitro* transcription
Sth1 sgRNA (T1)	GGGCGCTAAAGAGGAAGAGGACAGTTTTTGTACTCTCAAGATTCAA<br>TAATCTTGCAGAAGCTACAAAGATAAGGCTTCATGCCGAAATCAAC<br>ACCCTGTCATTTTATGGCAGGGTGTTTTCG	*In vitro* transcription
Sth1 sgRNA (T2)	GGGCCGGCGACGUUGGGUCAACUGTTTTTGTACTCTCAAGATTCAA<br>TAATCTTGCAGAAGCTACAAAGATAAGGCTTCATGCCGAAATCAAC<br>ACCCTGTCATTTTATGGCAGGGTGTTTTCG	*In vitro* transcription
Blat sgRNA (T1) direct	GGGCGCUAAAGAGGAAGAGGACAGCUAUAGUUCCUUACUGAAAGGU<br>AAGUUGCUAUAGUAAGGGCAACAGACCCGAGGCGUUGGGGAUCGCC<br>UAGCCCGUUUUUACGGGCUCUCCCCAUAUUCAAAAUAAUGACAGAC<br>GAGCACCUUGGAGCAUUUAUUUCCGAGGUGCUUUUUUUU	*In vitro* transcription
Blat sgRNA (T1) reverse	GGGCGCUAAAGAGGAAGAGGACAAUCAUAUCAUAUCGAGGAAACUU<br>GAUAUGAUAUGAUACUUUCAUUUUAUAUCCAUAUAUCAUCGAAGUC<br>AAUCUCAUUUAUCUGUCUAUUUUAUG	*In vitro* transcription
Blat sgRNA (T1) -3	GGGAAACGCTAAAGAGGAAGAGGGCUAUAGUUCCUUACUGAAAGGU<br>AAGUUGCUAUAGUAAGGGCAACAGACCCGAGGCGUUGGGGAUCGCC<br>UAGCCCGUUUUUACGGGCUCUCCCCAUAUUCAAAAUAAUGACAGAC<br>GAGCACCUUGGAGCAUUUAUUUCCGAGGUGCUUUUUUUU	*In vitro* transcription

Supplementary References

1. Stormo GD. **Modeling the specificity of protein-DNA interactions**. *Quant Biol.* 2013; **1**:115–130.

2. Crooks GE. **WebLogo: A Sequence Logo Generator**. *Genome Res.* 2004; **14**:1188–1190.

3. Markham NR, Zuker M. **UNAFold: software for nucleic acid folding and hybridization**. *Methods Mol Biol.* 2008; **453**:3–31.
